# Titrating lovaza from 4 to 8 to 12 grams/day in patients with primary hypertriglyceridemia who had triglyceride levels >500 mg/dl despite conventional triglyceride lowering therapy

**DOI:** 10.1186/1476-511X-11-143

**Published:** 2012-10-30

**Authors:** Charles J Glueck, Naseer Khan, Muhammad Riaz, Jagjit Padda, Zia Khan, Ping Wang

**Affiliations:** 1 Cholesterol Center, Jewish Hospital of Cincinnati, UC Health Building, 3200 Burnet Avenue, Cincinnati, OH 45229, USA

**Keywords:** Triglyceride, Lovaza, Triglyceride-lowering therapy, Severe primary hypertriglyceridemia

## Abstract

**Background:**

Omega-3 fatty acids are important in treatment of severe primary hypertriglyceridemia (HTG). In 15 patients with severe primary HTG (TG >500 mg/dl despite conventional TG lowering therapy), we assessed efficacy-safety of sequential monthly treatment with Lovaza, 4 to 8 to 12 g/day.

**Methods:**

With TG >500 mg/dl despite Type V diet, hyperinsulinemia and diabetes control, and fibric acids, Lovaza (4 g/d) was added for 1 month, and if TG remained >500 mg/dl, increased to 8 g/d for 1 month, and then to 12 g/d for 1 month, and subsequently reduced to 4 g/day for 4 months.

**Results:**

Primary HTG, median TG 884 mg/dl, 14 men, 1 woman, all white, age 50 ± 7 years, 12 non-diabetic, 3 with stable diabetes control. Weight and diet held stable throughout. In 5 patients, after 1, 2, and 3 months on 4 g/day, TG fell <500, mean 1390 to 234 (−83%, p<.0001), to 135 (−90%, p<.0001), and 158 mg/dl (−89%, p<.0001), with a negative TG slope, p=.0013. Non-HDLC fell from 320 to 177 (−45%, p=.001), to 152 (−53%, p=.0002), and to 163 (−49%, p=.0004), with a negative slope, p=.01. In 10 patients, with Lovaza increased from 4 to 8 to 12 g, 3 failed to respond. In 7 of these 10 patients, TG fell 37% from 1075 to 672 on 4 g (p=.006), to 577 on 8 g (−46%, p=.0009), and to 428 mg/dl (−60%, p<.0001) on 12 g/day, with a negative TG slope, p=.0018. TG on 12 g/day was lower than on 8 g/day, p =.03. Non-HDLC fell from 245 to 217 mg/dl (−11%) on 4 g/day, to 203 (−17%, p=.01) on 8 g/day, and to 192 (−22%, p=.003) on 12 g/day, with a negative slope, p=.016. Compared to pre-Lovaza baseline, no abnormal measures developed in safety tests. The 4, 8, and 12 g/d Lovaza doses were well tolerated.

**Conclusion:**

Titration of Lovaza from 4 to 8 to 12 g/d safely offers an effective way to lower TG beyond conventional 4 g therapy.

## Introduction

Omega-3 fatty acids of marine origin are safe, effective triglyceride (TG)-lowering agents [1-4], and are therapeutic in combined hyperlipidemia when added to statins [5]. Although the usual dose of the omega-3 marine long chain polyunsaturated fatty acid preparation (Lovaza) is 4 g/day, our previous studies [6-8] have shown that in patients receiving conventional triglyceride lowering regimens plus 4 g per day of Lovaza, further elevation of Lovaza to 8 g, and if necessary, to 12 g safely further lowers TG. Overall, there appears to be a generally linear decrease in TG with increasing dose of Lovaza [7]. In other studies of hypertriglyceridemic patients, intake of 4.5, 7.5, and 12 g omega-3 fatty acids have been used effectively over short treatment durations [9-12].

There are many patients with primary and familial hypertrigyceridemia (HTG) where conventional treatment with diet, avoidance of alcohol, fibric acids, diabetes control, metformin (for hyperinsulinemia) [13], and 4 g/day Lovaza fails to lower TG below 500 mg/dl, with TG levels remaining well above the therapeutic target [14] of 200 mg/dl [7]. In the Adult Treatment Panel-III guidelines, TG >500 was identified as severe, and a target for intervention, while an optimal TG level was <150 mg/dl [14]. To the best of our knowledge there is no published data which indicates that systematic stepwise increments of Lovaza to 8 or 12 g per day would have therapeutic effectiveness in further normalizing TG in subjects on conventional TG lowering regimens which include Lovaza 4 g/day. We hypothesized, based on our clinical experience [6-8], that increasing Lovaza to 8 and then (if necessary) to 12 g/day would safely further lower TG in subjects with severe primary HTG who failed to lower TG below 500 mg/dl on conventional therapy including Lovaza 4 g/day.

Our specific aim in the current study was to assess effectiveness and safety of Lovaza 4, 8, and 12 g/day in 15 patients who were referred to us with primary HTG (TG>1000 mg/dl) and failed to lower TG below 500 mg/dl despite treatment with diet, avoidance of alcohol, fibric acids, treatment of type 2 diabetes mellitus, and, in the presence of hyperinsulinemia, metformin [13].

## Results

There were 14 men, 1 woman, all white, mean age 50, baseline mean ± SD TG 1251 ± 671 mg/dl, with 25^th^, 50^th^ and 75^th^ percentile TG of 791, 884, and 1640 mg/dl, Table 1. Baseline mean ± SD HDL cholesterol was 29 ± 9 mg/dl and non-HDLC was 274 ± 104 mg/dl, Table 1.

**Table 1:** Baseline Lipids, lipoproteins, and APO E genotype in 15 patients with triglyceride >500 mg/dl

	**Mean ±SD**	**Percentile**		
		**25**^**th**^	**Median**	**75**^**th**^
Age (years)	50 ±7	44	50	54
TG (mg/dl)	1251 ± 671	791	884	1640
Total cholesterol (mg/dl)	303 ± 107	231	253	373
HDLC (mg/dl)	29 ± 9	24	27	36
Non-HDLC (mg/dl)	274 ± 104	200	228	347

APOE genotype	2-3	2-4	3-3	3-4
Responders n=12	2 (17%)	1 (8%)	6 (50%)	3 (25%)
Non-responders n=3	0	0	0	3 (100%)
Total n=15	2/15 (13%)	1/15 (7%)	6/15 (40%)	6/15 (40%)

APOE 3–4 was more common in non-responders, Fisher’s p=. 04.

Of the 3 subjects with type 2 diabetes, hemoglobin A1C (HbA1C) was held stable below 7.2% throughout the study period in 2, while in the third subject, mean HbA1C was 8.2%, but stable throughout the study. All patients with type 2 diabetes were treated with metformin 2.5 g/day. Of the 12 non-diabetic patients, 5 had impaired fasting glucose (≥ 100 mg/dl, 3 of these 5 had hyperinsulinemia), and another 5 patients had hyperinsulinemia (>24.9 uU/ml). All of these 10 patients were treated with branded Metformin 2.5 g/day.

Two subjects had ApoE 2–3 heterozygosity, 1 ApoE 2–4, 6 were homozygous for the wild-type normal genotype (ApoE 3–3), and 6 were ApoE 3–4 heterozygotes, Table 1. ApoE 3–4 was more common in non-responders (TG >500 mg/dl despite 12 g Lovaza), Fisher’s p=.04, Table 1. All 3 patients who failed to have sustained consistent reductions in TG below 500 mg/dl as Lovaza was increased from 4 to 8 to 12 g/day (non-responders) had the ApoE 3–4 genotype, Table 1.

**Figure 1:**
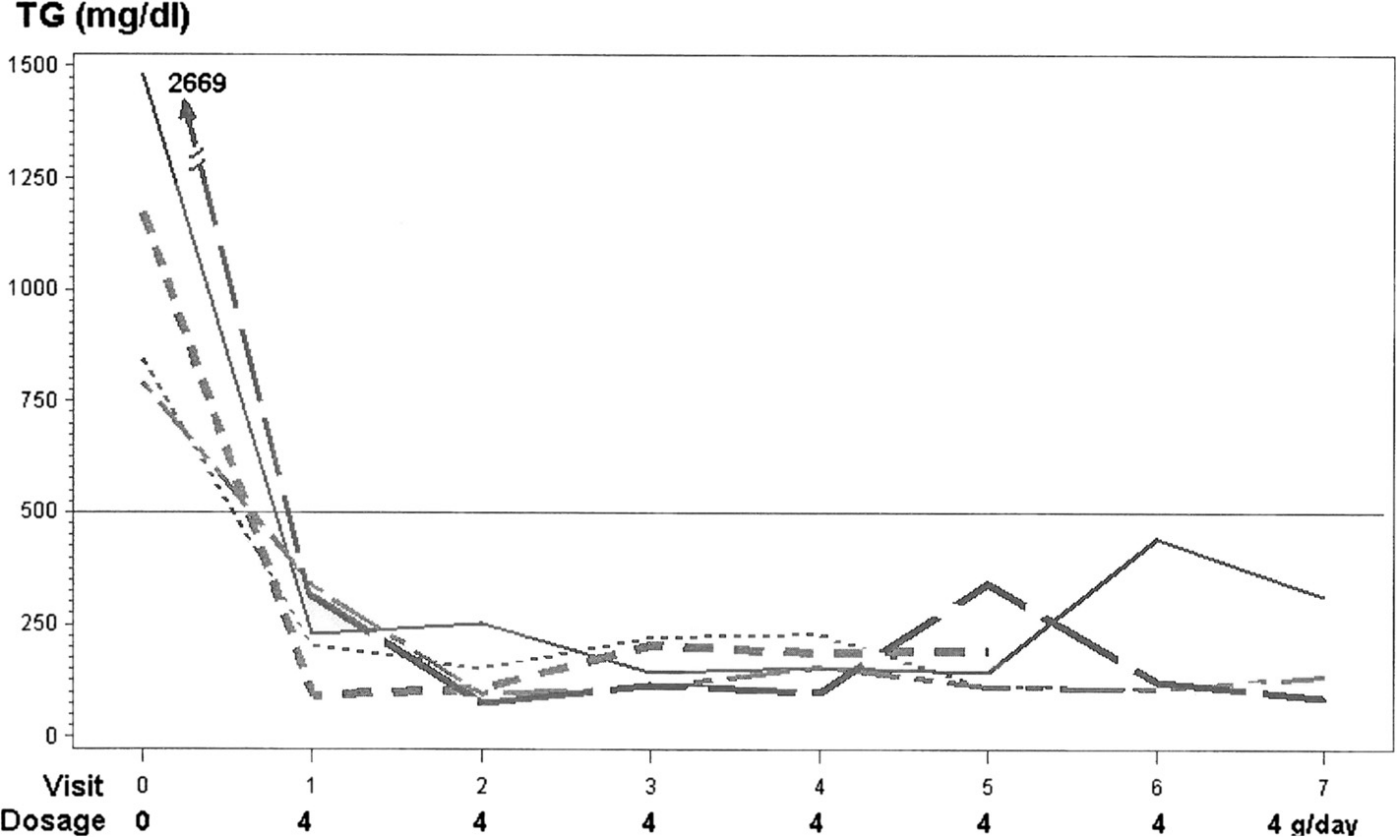
Five subjects whose triglyceride levels fell below 500 mg/dl on 4 grams of Lovaza/day.

After 1 month on 4 g/day Lovaza, in 5 patients, TG fell well below 500 mg/dl, from a baseline mean of 1390 mg/dl on conventional TG-lowering therapy to 234 mg/dl (mean reduced 83%, p<.0001), Figure 1, Table 2. Mean TG fell 90% to 135 mg/dl and to 158 mg/dl (−89%) after 2 and then 3 months on Lovaza 4 g/day (p<.0001 for both), with a negative slope as TG fell, p=.0013, Table 2. In this group of 5 patients, mean TG <200 mg/dl was maintained over 6 months treatment with 4 g/day Lovaza, Figure 1, Table 2. Mean non-HDL cholesterol fell from 320 to 177 mg/dl (decreasing 45%, p=.001) to 152 (−53%, p=.0002) to 163 mg/dl (−49%, p=.0004), Table 2. In these 5 patients, on 4 g/day Lovaza compared to baseline, there were no changes in body weight (p>.05), a single month period with lower systolic blood pressure (p=.04), and lower diastolic blood pressure (p=.05) in 2 of the 7 months on Lovaza, Table 3. In these 5 patients, there was no significant correlation between reduction in body weight (median 216 falling to 214) and reduction in TG (median 1170 mg/dl falling to 227 mg/dl) from visit 0 to visit 1, Spearman correlation r= −.22, p>0.5.

**Table 2:** TG and Non-HDLC at each visit during treatment in 5 subjects, where the Lovaza sequence was 4,4,4,4,4,4,4 g/day

**Visit**	**Dosage (g/day)**	**TG (mg/dl)**				**Non-HDLC (mg/dl)**			
		**Mean ± SD**	**median**	**% change from Visit 0**	**p (vs Visit 0)**	**Mean ± SD**	**median**	**% change from Visit 0**	**p (vs Visit 0)**
0	0	1390 ±766	1170			320 ±134	253		
1	4	234 ±97	227	−83%	<.0001	177 ±65	201	−45%	.001
2	4	135 ±71	107	−90%	<.0001	152 ±10	147	−53%	.0002
3	4	158 ±51	142	−89%	<.0001	163 ±19	171	−49%	.0004
4	4	164 ±48	155		<.0001	162 ±34	169		.0004
5	4	180 ±96	144		<.0001	170 ±44	151		.0007
6	4	194 ±166	114		<.0001	156 ±36	159		.0005
7	4	179 ±120	136		<.0001	139 ±30	148		.0004
		TG decreasing slope during the first 3 months, when dose changed from 0 to 4 g/day, p=.0013				Non-HDLC decreasing slope during the first 3 months, when dose changed from 0 to 4 g/day, p=.01			

**Table 3:** Body weight and blood pressure at each visit in 5 subjects, where the Lovaza sequence was 4,4,4,4,4,4,4 g/day

**Visit**	**Dose**	**Body weight (lb)**			**Systolic BP (mmHg)**			**Diastolic BP (mmHg)**		
		**Mean ± SD**	**median**	**p vs Visit 0**	**Mean ± SD**	**median**	**p vs Visit 0**	**Mean ± SD**	**median**	**p vs Visit 0**
0	0	209 ±51	216		126 ±19	120		86 ±14	80	
1	4	202 ±54	214		116 ±15	110		75 ±11	80	.05
2	4	202 ±51	214		110 ±7	110	.04	77 ±8	80	
3	4	199 ±49	211		116 ±14	110		78 ±13	76	
4	4	199 ±50	209		121 ±17	130		74 ±10	78	.05
5	4	202 ±51	214		122 ±13	118		78 ±5	80	
6	4	201 ±60	225		122 ±2	122		77 ±6	80	
7	4	201 ±59	224		120 ±12	116		77 ±6	77	
**In 7 subjects, where the Lovaza sequence was 4, 8, 12, 4, 4, 4,4 g/day**										
**Visit**	**Dose**	**Body weight (lb)**			**Systolic BP (mmHg)**			**Diastolic BP (mmHg)**		
		**Mean ± SD**	**median**	**p vs Visit 0**	**Mean ± SD**	**median**	**p vs Visit 0**	**Mean ± SD**	**median**	**p vs Visit 0**
0	0	251 ±30	246		138 ±28	124		88 ±13	86	
1	4	251 ±31	246		139 ±25	132		88 ± 9	86	
2	8	253 ±28	247		129 ±23	124		85 ±18	78	
3	12	250 ±29	242		122 ±10	122		84 ±17	80	
4	4	250 ±31	242		121 ±12	120	.05	79 ± 8	80	.05
5	4	251 ±29	249		130 ±16	130		85 ±12	82	
6	4	247 ±33	244		136 ±24	132		83 ±14	80	
7	4	243 ±34	235	.002	132 ±24	124		78 ±12	76	.02
**In 3 non-responders, where the Lovaza sequence was 4, 8, 12, 4, 4, 4, 4 g/day**										
**Visit**	**Dose**	**Body weight (lb)**			**Systolic BP (mmHg)**			**Diastolic BP (mmHg)**		
		**Mean ± SD**	**median**	**p vs Visit 0**	**Mean ± SD**	**median**	**p vs Visit 0**	**Mean ± SD**	**median**	**p vs Visit 0**
0	0	204 ±10	204		121 ±17	120		84 ±4	85	
1	4	202 ±11	196		115 ±15	110		77 ±7	76	
2	8	198 ±14	191		122 ±3	120		87 ±6	90	
3	12	200 ±14	195		125 ±8	120		82 ±5	84	
4	4	198 ±13	192		113 ±12	120		76 ±4	75	
5	4	196 ±12	190	.02	126 ±14	134		79 ±10	77	
6	4	204 ±11	204		129 ±1	129		82 ±0	82	
7	4	202 ±19	202		123 ±24	123		82 ±11	82	

**Figure 2:**
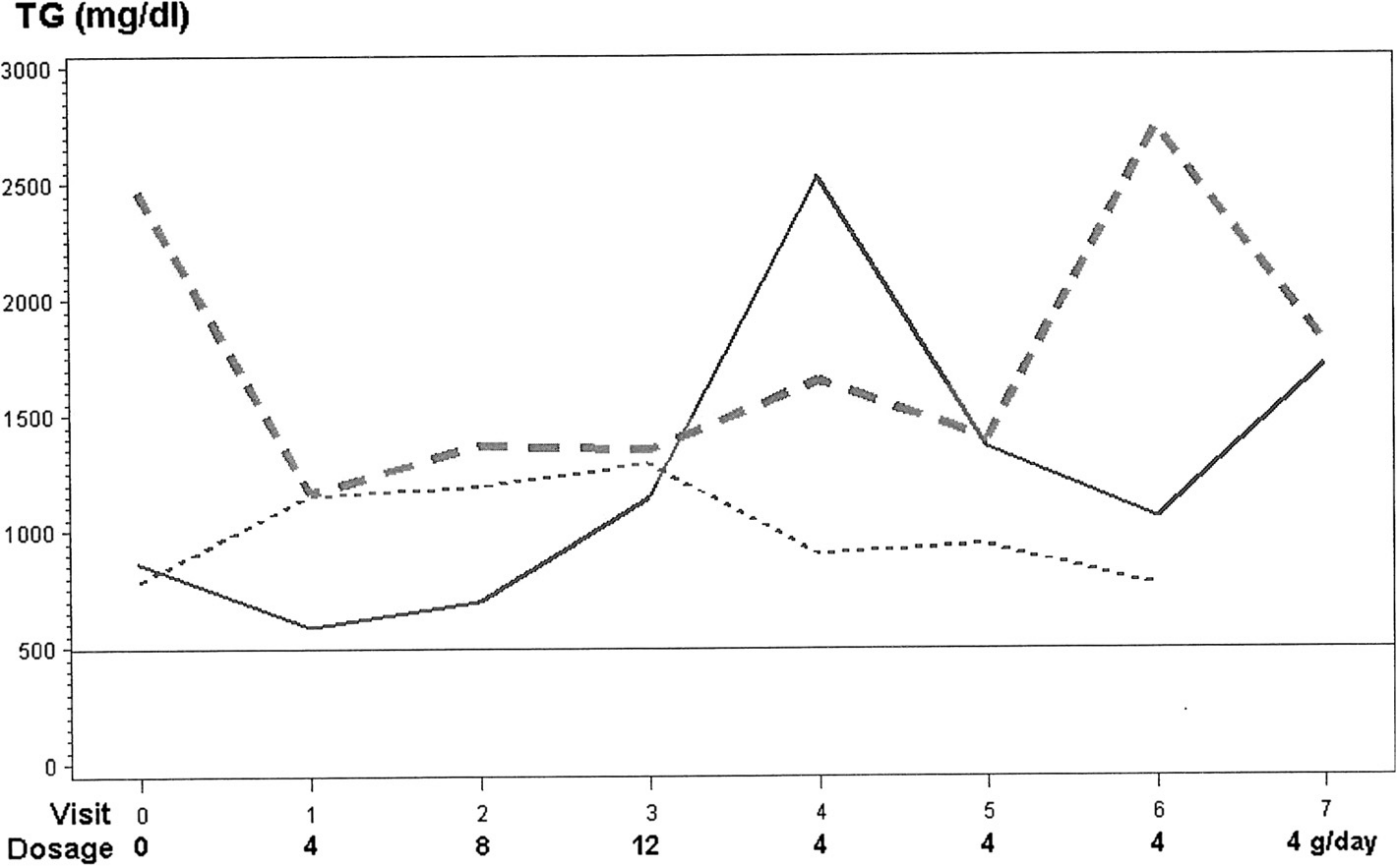
Three subjects whose triglyceride levels failed to fall below 500 mg/dl despite titration of Lovaza from 4 to 8 to 12 grams/day.

Of the 10 patients (baseline mean TG 1163 mg/dl on conventional TG-lowering therapy), as Lovaza was increased from 4 to 8 to 12 g/day, 3 failed to consistently respond, with mean TG at baseline 1369 mg/dl, and after 3 months 1260 mg/dl while on Lovaza 12 g/day, Figure 2, Table 4. One of these three non-responders was the subject with type 2 diabetes whose mean HbA1C was stable at 8.2% throughout the study. In this subject, TG fell from 859 mg/dl at baseline to 631 mg/dl on 4 g/day, rose to 697 on 8 g/day, and rose further to 1141 mg/dl on 12 g/day, Figure 2. Then, when Lovaza was reduced to 4 g/day, TG rose to 2530 mg/dl, Figure 2. In this group of 3 patients, body weight was lower (p=.02) at one of the 7 visits on Lovaza, without significant changes in systolic or diastolic blood pressure, Table 3.

**Table 4:** TG and Non-HDLC at each visit during treatment In 3 non-responders, where the Lovaza sequence was 4, 8,12, 4, 4, 4, 4 g/day

**Visit**	**Dosage (g/day)**	**TG (mg/dl)**			**Non-HDLC (mg/dl)**		
		**Mean ± SD**	**median**	**p vs Visit 0**	**Mean ± SD**	**median**	**p vs Visit 0**
0	0	1369 ±950	859		256 ±79	221	
1	4	970 ±328	1154		258 ±121	229	
2	8	1086 ±348	1190		235 ±86	199	
3	12	1260 ±108	1290		242 ±121	211	
4	4	1693 ±816	1650		301 ±85	347	
5	4	1233 ±255	1360		248 ±86	237	
6	4	1522 ±1065	1060		263 ±51	266	
7	4	1760 ±71	1760		246 ±42	245	

**Figure 3:**
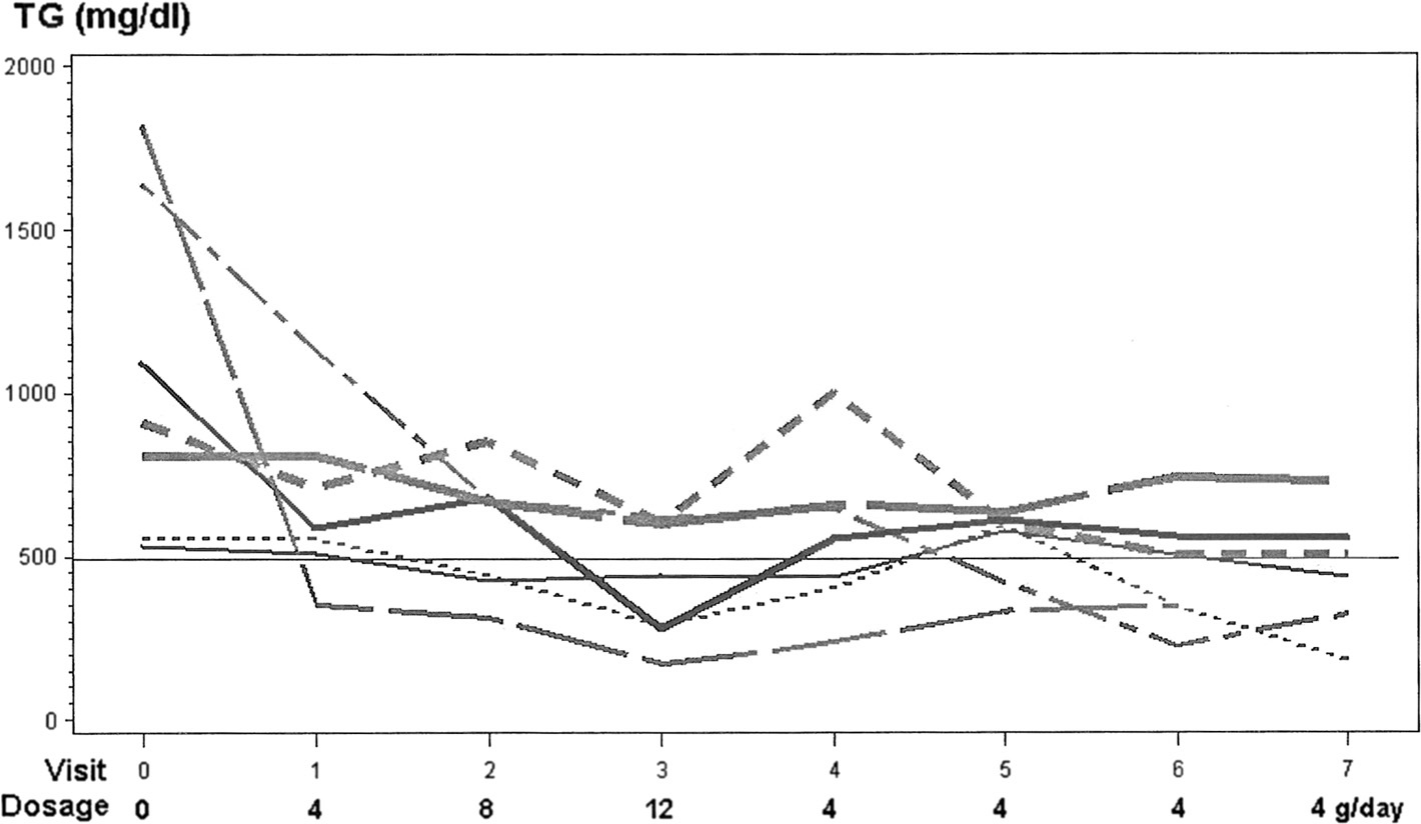
Seven subjects whose triglyceride levels fell as Lovaza was titrated from 4 to 8 to 12 grams/day.

In 7 patients who responded with TG lowering as Lovaza was increased to 12 g/day, Figure 3, Table 5, TG fell from a baseline mean on conventional TG lowering therapy of 1075 to 672 mg/dl on Lovaza 4 g/day (decreasing 37%, p=.006), to 577 on 8 g/day (46%, p=.0009), and to 428 mg/dl on 12 g/day (60%, p <.0001), Table 5, with a negative TG slope, p=.0018, Table 5. At the same time, mean non-HDLC fell 11% from 245 to 217 mg/dl, fell 17% to 203 mg/dl (p=.01), and fell 22% to 192 mg/dl (p=.003), Table 5. In this group of 7 patients, body weight was lower (p=.002) at the last of 7 visits on Lovaza, systolic blood pressure was lower in one visit (p=.05), and diastolic blood pressure lower at 2 visits (p=.05, p=.02), Table 3. In 3 of these 7 patients after 1 month on 8 g/day Lovaza, TG levels had fallen to <500 mg/dl (to 440, 422, and 371 mg/dl), but their Lovaza was increased to 12 g/day, rather than being retained for 1 month at 8 g/day.

**Table 5:** TG and Non-HDLC in 7 subjects, where the Lovaza sequence was 4, 8, 12, 4, 4, 4, 4 g/day

**Visit**	**Dosage (g/day)**	**TG (mg/dl)**				**Non-HDLC (mg/dl)**			
		**Mean ± SD**	**median**	**% change, p vs Visit 0**	p* (vs former Visit)	**Mean ± SD**	**median**	**% change, p vs Visit 0**	p* (vs former Visit)
0	0	1075 ±525	857			245 ±88	214		
1	4	672 ±247	587	−37%, .006		217 ±62	223	−11%,	
2	8	577 ±189	666	−46%, .0009	NS	203 ±44	199	−17%, .01	NS
3	12	428 ±184	443	−60%, <.0001	.03	192 ±61	164	−22%, .003	NS
4	4	561 ±244	554	.0006	.03	203 ±49	185	.01	NS
5	4	538 ±115	593	.0004		205 ±44	233	.01	
6	4	459 ±171	502	<.0001		189 ±31	185	.001	
7	4	430 ±191	395	<.0001		171 ±43	164	.0002	
		TG decreasing slope during the first 3 months, when the Lovaza dose was increased from 0 to 12 g/day, p=.0018				Non-HDLC decreasing slope during the first 3 months, when the Lovaza dose was increased from 0 to 12 g/day, p=.016			

p: comparison of Least-square means using Mixed model.

p*: paired Wilcoxon test for the change from Visit 1 to Visit 2 (dosage from 4 g/day to 8); from Visit 2 to Visit 3 (dosage from 8 g/day to 12); from Visit 3 to Visit 4 (dosage from 12 g/day to 4).

Of the 12 subjects without type 2 diabetes, 5 of whom had impaired fasting glucose (3 of these 5 had hyperinsulinemia), and 5 had hyperinsulinemia without IFG. All 10 patients were treated with metformin 2.5 g/day. Changes in weight, insulin and glucose were not significant (all p>0.6) from visit 0 to visit 1, and there was no correlation between the changes in insulin, weight, and TG from visit 0 to visit 1, p >0.6.

In the 15 patients, there were 85 patient-months on Lovaza 4 g/day, 10 patient- months on 8 g/day, and 10 patient-months on 12 g/day. Compared to pre-Lovaza baseline, no abnormal measures developed in laboratory safety tests (p>0.1) on 4, 8, or 12 g/day Lovaza therapy. The 4, 8, and 12 g/d Lovaza doses were well tolerated, and adherence by pill count was >95% for all treatment groups.

## Discussion

In the current study, after treating HTG with a conventional TG-lowering regimen (fibric acids, diet, diabetes control, and metformin, when hyperinsulinemic), 5 patients were very sensitive to addition of 4 g Lovaza/day, with mean TG falling 90% from 1390 mg/dl to 135 mg/dl and mean non-HDLC falling 53% from 320 to 152 mg/dl in 2 months. Moreover, at 2 of 7 monthly visits on Lovaza, diastolic blood pressure was lower than at baseline, an additional beneficial outcome.

In 10 patients with mean TG 1163 mg/dl despite conventional TG-lowering therapy, 3 patients (baseline mean TG 1369 mg/dl) failed to respond significantly to sequential increments of Lovaza from 4 to 8 to 12 g/day. In 7 of these 10 patients, however, TG fell 37% from 1075 mg/dl to 672 mg/dl on Lovaza 4 g/day, fell 46% to 577 mg/dl on 8 g/day, and fell 60% to 428 mg/dl on 12 g/day, with a negative TG slope. At the same time, mean non-HDLC fell 11% from 245 to 217 mg/dl, 17% to 203 mg/dl, and 22% to 192 mg/dl. Moreover, diastolic blood pressure was lower than baseline at last visits on Lovaza.

Patients with the ApoE 3–4 genotype were less responsive to Lovaza therapy, congruent with the report by Christidis et al. [15] where triglyceride reduction on fenofibrate was less marked in patients with the ApoE4 allele.

Titration of the Lovaza to 8 g/day (10 patient-months) and 12 g/day (10 patient- months) in 10 patients did not adversely affect any laboratory safety tests, did not produce petechiae or purpura, and was well tolerated. This is a limited experience, however, with regard to safety of 8 and 12 g/day of Lovaza, and is a limitation of the current study.

Another limitation of the current study was the failure to measure plasma, red blood cell, and leukocyte fatty acid profiles and compare these with the patients’ responses to the stepwise increases in doses of Lovaza.

Our findings in the current study are congruent with previous studies in hypertriglyceridemic patients given up to 15 g/day of omega-3 fatty acids [6-12]. Harris et al. [9] gave 4.5,7.5, and 12 g/d of omega-3 fatty acids for 3 successive 6 week periods to 10 hypertriglyceridemic patients, and triglycerides fell from 540 to 257 to 248 to 208 mg/dl as the dose increased, p<.01 versus baseline for each. In 22 women with median entry triglycerides 1087 mg/dl, Goldenberg et al. [7] safely titrated omega-3 fatty acids up to 12 g/day, with triglycerides falling to a median of 326 mg/dl. In 4 women whose initial triglyceride levels were >750 mg/dl despite conventional triglyceride-lowering therapy, Glueck et al. [8] safely titrated omega-3 fatty acids up to 15 g/d. Sanders et al. [10] safely treated 5 patients with primary hypertriglyceridemia with 15 g/d of omega-3 fatty acids over a 4 week period with mean triglyceride levels falling from 1147 to 558 mg/dl.

A triglyceride concentration above 1,000 mg/dl is an indication for therapy to lower the risk of acute pancreatitis, while in subjects with TG levels below 1,000 mg/dl the indication for treatment is primarily to reduce the risk of clinical events caused by atherosclerosis [7,8,16-18]. The relationship between hypertriglyceridemia and coronary heart disease is abolished when non-HDL cholesterol and HDL cholesterol concentrations are controlled for [19].

Omega-3 fatty acids have successfully been used in treatment of severe primary HTG [7,20,21]. Omega-3 fatty acids and fenofibrate have similar TG-lowering effects and promote similar changes in endothelium-dependent dilation, but fenofibrate therapy has substantially better effects on lipoprotein and metabolic profiles in patients with HTG [22]. In severe HTG, fibric acids and omega-3 fatty acids are often used concurrently [7,8,23,24]. In the current study, we did not add nicotinic acid to the entry conventional TG lowering regimen (low fat diet [<25% of calories as fat], fibric acids, metformin reduction of insulin resistance, diabetes control) to avoid worsening [25-28] of type 2 diabetes, conversion of impaired fasting glucose to levels ≥126 mg/dl, or worsening insulin resistance-hyperinsulinemia in patients with antecedent hyperinsulinemia.

Bays et al. [21] carried out a placebo-controlled randomized study of 229 diet-stable patients with fasting TG ≥ 500 mg/dl and ≤ 2,000 mg/dl. Mean baseline TG levels were 680, 657, and 703 mg/dl for eicosapentaenoic acid ethyl ester (AMR101) 4 g/day, AMR101 2 g/day, and placebo. AMR101 4 g/day reduced the placebo-corrected TG levels by 33.1% (p <.0001) and AMR101 2 g/day by 19.7% (p =.0051). For patients with baseline TG levels >750 mg/dl, AMR101 4 g/day reduced the placebo-corrected TG levels by 45.4% (p =.0001) and AMR101 2 g/day by 32.9% (p =.0016). AMR101 significantly reduced non-HDLC.

Another approach to the stepped management of the type of severely hypertriglyceridemic patients described in this report would be to lower TG to < 1,000 mg/dL with Lovaza, and then add atorvastatin, 80 mg/day, or rosuvastatin, 40 mg/day [29].

## Conclusion

In patients with severe primary HTG despite conventional TG lowering therapy, addition of Lovaza 4 g per day, and where necessary, increasing to 8 or 12 g per day safely further lowers TG in a linear fashion.

## Methods

### Study design, patients

The study followed a protocol approved by the Jewish Hospital IRB, with signed informed consent, and after an entry visit, included 8 subsequent visits at monthly intervals. Patients were instructed in a eucaloric diet with total fat restricted to no more than 25% to 30% of calories [30] at the entry visit by registered dietitians, with no alcohol intake. At each monthly visit, dietary adherence was reviewed by the dietitians, using 24-hour recall. Weight loss was not a targeted goal during the 8-month treatment period.

### Inclusion criteria

Inclusion criteria included primary HTG [18] with fasting TG levels >1000 mg/dl, and persistence of TG levels > 500 mg/dl despite conventional TG lowering therapy for 1 month, including diet, control of type 2 diabetes mellitus, fibric acids, and, where indicated, metformin for treatment of hyperinsulinemia. Type 1 diabetics were excluded; three type 2 diabetics with stable hemoglobin A1C control were included.

### Exclusion criteria

1. Patients with known allergy to fish.

2. HTG secondary to alcoholism, exogenous corticosteroids or estrogens, nephrotic syndrome, hemochromatosis, glycogen storage disease, uncontrolled diabetes, Cushing’s syndrome, uremia.

3. Bleeding gastric or duodenal ulcers, active inflammatory bowel disease.

4. Pregnancy.

5. Dementia.

After an overnight fast, blood was obtained for entry measurement of lipid profile, complete blood count, fasting glucose, hemoglobin A1C (HbA1C), insulin, C-peptide, renal and liver function tests, T4 and TSH, and apo E genotype. At each of 8 subsequent visits, blood was drawn for lipid profile, complete blood count, glucose, HbA1C, renal and liver function, insulin, and C peptide. At entry, and at each subsequent visit, dermatologic examinations were carried out to record any petechiae or bruising. At entry, and at 8 subsequent visits, weight and blood pressure were measured, and a brief physical exam was carried out. At each visit, adherence to the targeted Lovaza dose was assessed by pill count, and adherence to diet by a 24-hour diet recall.

### Lovaza Dose Schedule, serial monthly visits

#### Visit −1 (Entry)

Patients referred to our center with TG >1,000 mg/dl were instructed in a eucaloric diet with total fat restricted to no more than 25% to 30% of calories [30], with alcohol restricted to zero. If the patient was not already taking fenofibrate 145 mg/day or gemfibrozil 1.2 g/day, these therapies were instituted. All the 3 patients with type 2 diabetes received metformin 2.5 g/day, and one (with HbA1C >7.2%) also received liraglutide 1.8 mg/day. Nicotinic acid was not used since many of the patients either had type 2 diabetes mellitus, or impaired fasting glucose, or hyperinsulinemia which were relative contraindications [25-28] to its use. Patients with impaired fasting glucose and/or fasting hyperinsulinemia (>24.9 uU/ml) were also treated with metformin [31,32], 2.5 g/day.

### Visit 0 (Baseline)

One month after visit −1, if TG remained >500 mg/dl despite conventional TG lowering therapy, then patients were instructed to start Lovaza, 2 g with breakfast, 2 g with the evening meal, while continuing their antecedent TG lowering therapy unchanged, and dietary instruction was reinforced.

### Visit 1

One month after visit 0, if TG had fallen to < 500 mg/dl, Lovaza was held at 4 g/day. If TG remained >500 mg/dl, Lovaza was increased to 8 g/day, with other components of the TG lowering regimen held stable throughout.

### Visit 2

One month after visit 1, if TG had fallen to < 500 mg/dl on 8 g/day, the Lovaza dose was kept at 8 g/day, but if TG remained > 500 mg/dl, Lovaza was increased from 8 to 12 g per day, with other components of the TG lowering regimen held stable.

### Visit 3

One month after visit 2, Lovaza was diminished in all patients to 4 g/day.

### Visits 4–7

Four monthly visits after visit 3; Lovaza kept at 4 g/day.

### Statistical analysis

All statistical analysis was done using SAS 9.1.3.

For each treatment group (according to Lovaza dosage sequences), mean ± SD and median TG, non-HDLC, body weight and blood pressure (BP) at each visit were calculated. Changes from baseline to follow-up were tested using MIXED model, with visit as fixed effect, subject as random effect, and comparison of least square means (SAS proc MIXED).

For each treatment group, MIXED model for TG was constructed for the first 3 months on Lovaza, fixed effect was time (visit order), random effect was subject, with repeated measures had AR(1) variance structure. Using MIXED model, the TG slope was assessed.

Paired Wilcoxon tests were used to assess the effect of changes of Lovaza dosage (from 4 to 8 g/day, from 8 to 12 g/day, from 12 to 4 g/day) on TG and non-HDLC.

## Abbreviations

(HTG): Primary hypertriglyceridemia; (TG): triglyceride; (HDLC): high density lipoprotein cholesterol; (non-HDLC): non high density lipoprotein cholesterol.

## Competing interests

Beyond provision of support by way of drug and partial funding by GSK, the authors have no conflict of interest, including membership in speaker’s bureau or corporate advisory committee, stockholder, or travel support. No conflicts of interest with competing products.

## Authors’ contributions

Drs NK, CJG, and PW participated in all aspects of the manuscript, study design, data collection, data editing, data analysis, production of manuscript. Drs ZK, MR, and JP participated in data collection, data editing, data analysis, production of manuscript. All authors read and approved the final manuscript.
